# Differences in the absolute muscle strength and power of children and adolescents with overweight or obesity: a systematic review

**DOI:** 10.1186/s12887-023-04290-w

**Published:** 2023-09-19

**Authors:** José Luis Alaniz-Arcos, Ma. Elena Ortiz-Cornejo, José Omar Larios-Tinoco, Miguel Klünder-Klünder, Karla Vidal-Mitzi, Claudia Gutiérrez-Camacho

**Affiliations:** 1 Physiotherapy Research Unit, Faculty of Medicine, Universidad Nacional Autónoma de México, Dr. Márquez 162 Colonia Doctores, Mexico City, CP 06720 Mexico; 2 Research Headmaster’s Office, Hospital Infantil de México Federico Gómez, Mexico City, México

**Keywords:** Absolute muscle strength, Power, Assessment instruments, Muscle tests, Obesity, Overweight

## Abstract

This study aimed to describe absolute muscle strength and power in children and adolescents with obesity, overweight and normal weight, and the assessment tests and tools used. We retrieved observational studies from MEDLINE (PubMed), TripDataBase, Epistemonikos, EBSCO essentials, NICE, SCOPUS, and LILACs up to February 2023. In addition, we recovered data from studies with at least three comparison groups (obesity, overweight, normal weight) and with a description of the absolute muscle strength and power and the assessment tests and instruments used. The methodologic quality of the studies was assessed with the Joanna Briggs checklist, and the review was carried out using the PRISMA 2020 methodology. Eleven studies with 13,451 participants from 6 to 18 years of age were once included, finding that the absolute muscle strength of their upper extremities was greater when they were overweight or obese; however, in the same groups, absolute muscle strength was lower when they carried their body weight. In addition, lower limb absolute muscle strength was significantly lower in obese participants than in normal weight, regardless of age and gender. The most used tools to measure the absolute muscle strength of the upper limbs were the grip dynamometers and push-up exercises. In contrast, different jump tests were used to measure the power of the lower limbs. There are great differences in muscle strength and power between overweight or obese children and adolescents and those with normal weight. Therefore, it is recommended to use validated tests, preferably that assess strength through the load of the patient’s body weight, either of the upper or lower limbs, for greater evaluation objectivity that facilitates the management of these children and adolescents.

**Supplementary Information:**

The online version contains supplementary material available at 10.1186/s12887-023-04290-w.

## Background

Obesity and overweight are global health problems that affect children and adolescents [1, 2]. In 2020, the World Health Organization reported that 39 million children under five years of age and over 340 million children and adolescents between 5 and 19 years lived with overweight or obesity [3].

Obesity and overweight have been associated with several adult diseases, leading to disability and decreased quality of life [4]. Besides, there are reports in the literature that have described a higher prevalence of musculoskeletal disorders in overweight or obese children compared to children with a normal weight (16% vs. 14.1%; OR 95% CI = 1.16 (0.84–1.61) [5]. The above has been explained because visceral adipose tissue hypertrophy contributes to muscle dysfunction, mainly through the dysregulated production of adipokines; for this reason, the muscle cell is less efficient when executing signaling functions [6]. Obesity also increases the secretion of adiponectin, and the production of inflammatory mediators, while decreasing the synthesis of contractile proteins in the myotubes of muscle fibres [7].

Furthermore, there is growing evidence showing the effects of obesity on skeletal muscle function, such as [8] impairment in oxidative capacity, [9] abnormal muscle fibre organization, [10] interrupting the calcium cycle, [11] inducing easy fatigue, [7] and a decline in contractile function [12] and the change of slow-twitch fibres to fast-twitch ones [13].

On the other hand, clinical studies on the effects of obesity on muscle size and function have shown that muscle torque, and power, in people with obesity, are higher than those of normal weight [14]. Obesity has also been associated with reduced maximum muscle strength, affecting mainly the function of antigravity muscles, leading to reduced mobility [15].

Additionally, muscle strength is considered an essential factor in the development of children and adolescents, which is necessary to carry out daily life activities such as self-care, walking, or running, which are essential to facilitate adequate social interaction and prevent diseases in adulthood [16–19].

Even though the evaluation of muscle strength is necessary due to the above, most of the studies in children and adolescents are carried out with tests used in adults, which are “adapted” and rarely validated, which makes it difficult to know precisely the degree of impairment of muscle strength and power in overweight and obese children. Therefore, the purpose of this study was to show the most used tests to assess muscle strength and power in patients living with overweight and obesity, which provides valuable information for the personnel responsible for managing these patients.

## Materials and methods

### Data sources

This review was carried out following the question frame PEOS (Patient, Exposure, Outcome, Study): P = Participants aged 6–18 years, E = obesity or overweight or normal weight, O = muscle strength, muscle power, and S = Observational studies. This review was not registered in any international database of prospectively systematic reviews, but it was carried out through the PRISMA methodology. An additional file shows the description of the assessment test considered in this review; see Supplementary Material Annex 1.

**Fig. 1:**
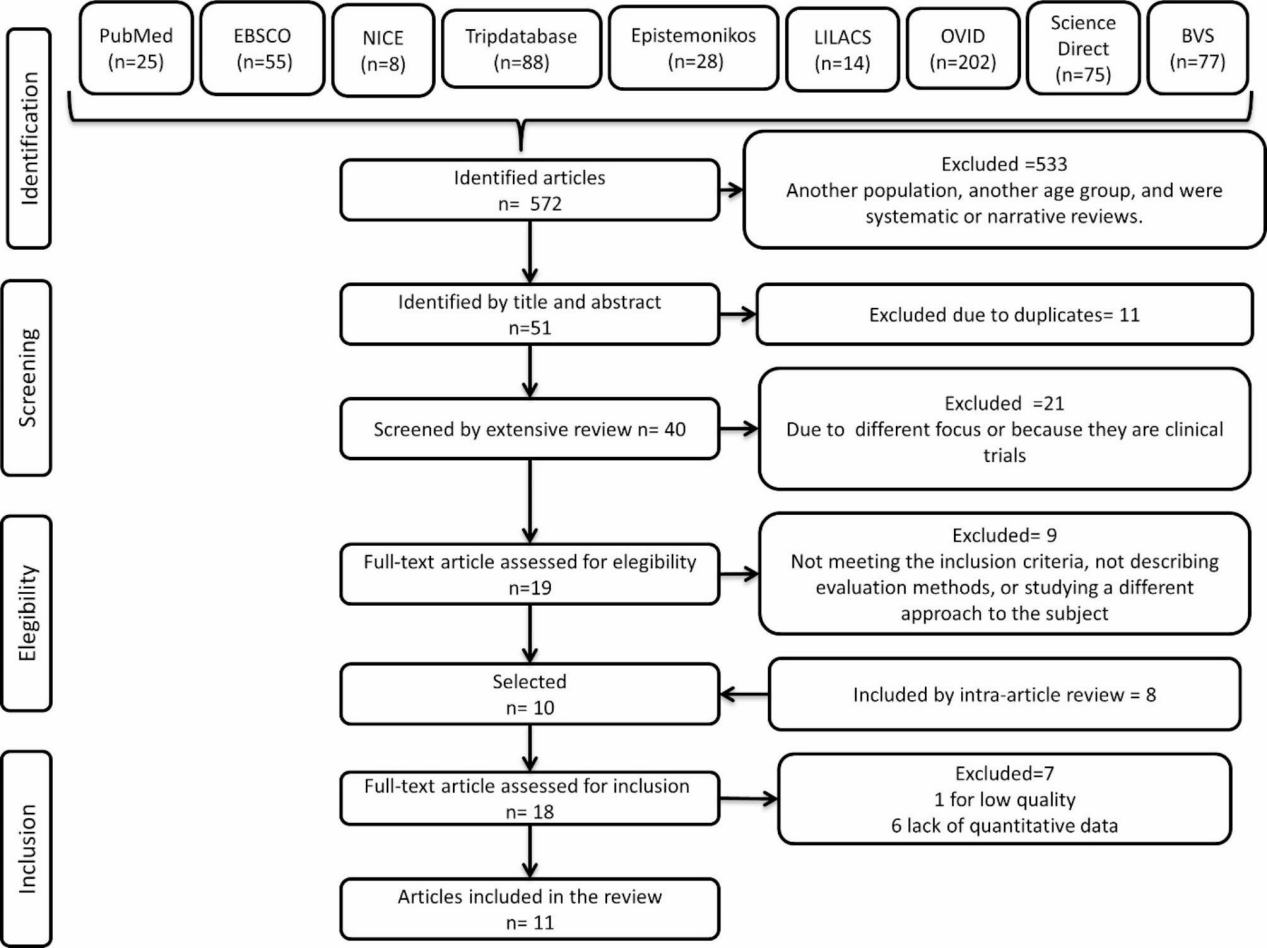
PRISMA flow chart

Initially, we developed different search strategies in the electronic database MEDLINE (PubMed) using the MeSH terms: “child,” “obesity,” “muscle strength,” and ”physical fitness,” and through the search process, resulted in this final search strategy: “overweight AND obesity AND child AND muscular AND perform AND muscle strength AND physical fitness AND fitness,” and then we adapted this strategy to the different search engines; TripDataBase, Epitemonikos, EBSCO essentials, NICE, SCOPUS, OVID, ScienceDirect, BVS and LILACs (Fig. 1). An additional file shows the search strategy used to identify studies; see Supplementary Material Annex 2.

### Selection of studies

Inclusion criteria; Observational studies carried out in overweight or obese children or adolescents, in which the strength and muscular power were evaluated, and the tests and instruments for their measurement were included. We also included studies written in English and Spanish with at least two comparison groups; obese vs. non-obese, obese vs. overweight, or more groups; underweight, normal weight, overweight, and obesity. Exclusion criteria; Studies that did not allow differentiating the results between the comparison groups were excluded. Studies in patients with endogenous causes of their obesity and studies without a detailed description of the groups evaluated were also excluded. Selection process; Firstly, the titles and abstracts of identified studies were independently reviewed by five authors (JLAA, MKK, MEOC, JOLT, KVM). Secondly, the full text of each study deemed possibly relevant was retrieved and independently reviewed by the same five authors. Each review author prepared a list of studies that they believed met the inclusion criteria, and then all of them were compared for the final selection. Any disagreements were resolved by discussion and consensus with a sixth author (CGC). The Mendeley Reference Manager 2.95.0 was used as the software to remove duplicate articles. Finally, once the review of titles and abstracts by the reviewers was completed, the full texts were reviewed, and the reasons for being chosen were recorded. We performed another search within the references of the articles selected to identify additional studies. The searching strategy was updated in February 2023, and four more studies were identified by title and abstract but were excluded due to; a different approach; they did not report muscle strength and power data.

### Data extraction

The data extraction and management from the included studies adhered to the recommendations of the PRISMA 2020 expanded checklist [20]. Five review authors (JLAA, MKK, MEOC, JOLT, KVM) extracted data using specially developed data extraction Excel sheets. The information extracted from each study was the following: participants’ age, sex, country, a sample size of the comparison groups (obese vs. no obese, obese vs. overweight, obese vs. overweight vs. normal weight); assessment tests and instruments used and parameters evaluated such as muscle strength, muscle power, physical activity level, balance, cardiovascular capacity, coordination, flexibility, agility, velocity, although the main focus was on the absolute muscle strength and power data. We defined muscular strength as the force generated by muscle contraction and can be measured during isometric, isotonic, or isokinetic contraction. Muscular power was defined as the work rate, reflecting the force exerted per unit of time [21].

#### Quality assessment

We used the Joanna Briggs Institute Critical Appraisal tools in JBI Systematic Checklist for Prevalence Studies (JB checklist) to identify the methodological compliance of the selected articles [22]. This checklist was applied independently by five raters (JLAA, KVM, MOEC, JOLT, MKK) to be later verified by a sixth rater (CGC), who resolved the conflicts. The JB checklist was intended to assess the methodological quality of a study and determine whether there was bias in design, conduct, and analysis.

The results of this evaluation were used to inform compliance with items such as; Yes, No, Uncertain, or Not applicable (Table 1). Where studies did not meet most checklist items or the information needed to be more specific, they were excluded.

**Table 1:** Quality assessment of the articles included

Reference /ítem	Appropriate sample frame	The appropriate way for the sample	Appropriate sample size	Study subjects	Data analysis coverage	Valid methods to assess the condition	A standard and reliable way to measure the condition	Appropriate statistical analysis	Response rate and its management
Lazzer, S. et al., 2009	U	U	U	U	U	Yes	Yes	Yes	Yes
Riddiford-Harland, DL. et al., 2006	No	U	U	No	U	Yes	Yes	Yes	Yes
He, H. et al., 2019	Yes	Yes	Yes	Yes	Yes	Yes	Yes	Yes	Yes
Ervin, RB. et al., 2014	Yes	Yes	Yes	No	Yes	Yes	Yes	Yes	Yes
Karppanen, AK. *et a,l* 2012	Yes	U	U	Yes	No	Yes	Yes	Yes	U
Nunez-Gaunaurd, N. et al., 2013	Yes	U	U	Yes	Yes	Yes	Yes	Yes	Yes
Deforche, B. et al., 2003	Yes	Yes	Yes	Yes	Yes	Yes	Yes	Yes	Yes
Tokmakidis, S. et al., 2006	Yes	Yes	Yes	Yes	Yes	Yes	Yes	Yes	Yes
Sachetti, R. et al., 2012	Yes	Yes	Yes	Yes	Yes	Yes	Yes	Yes	Yes
Castro-Piñero, *J. et al.*, 2012	Yes	Yes	Yes	Yes	Yes	Yes	Yes	Yes	Yes
Ceschia, A. et al., 2015	Yes	Yes	Yes	Yes	Yes	Yes	Yes	Yes	Yes

Yes= Well described; No= Not described; U= Uncertain. Joanna Briggs Institute (JBI) critical appraisal tools for use in JBI Systematic Reviews

#### Data analysis

Data from all included studies were summarised and described. We did not compare their results because we considered that the publication bias could have been higher in most of the studies by not complying with the Joanna Briggs checklist. The clinical tests to measure muscle strength and power used in the included studies were not similar enough, so we considered it unsuitable to perform a meta-analysis.

## Results

A total of 572 articles were identified in the electronic databases shown in Fig. 1. We excluded 533 articles because muscle strength and power data were not described, participants had muscular or genetic disorders, the focus of the study was different, or it was published in languages other than English or Spanish. Fifty-one articles were selected by title and abstract; 11 were eliminated due to being duplicated in the electronic databases consulted. Subsequently, the remaining 40 were reviewed entirely, resulting in the exclusion of 21 since the focus of the study was different and needed to describe the variables of interest; the other nine were excluded from the 19 remaining articles due to not describing evaluation methods or studying participants with a different approach. Subsequently, ten articles were selected. Therefore, we performed an intra-article review of the ten remaining articles and identified eight more articles; however, six were eliminated due to low quality (JB checklist) and the other one because of a lack of quantitative data. Finally, 11 articles were included in this work. (Fig. 1) The Kappa coefficient was calculated to assess the agreement between the raters, whose value was 0.82 (p ≤ 0.001) with a percentage agreement of 92%.

The 11 included articles were carried out between 2003 and 2023, with a total of 13,451 participants, ​ ​of which seven were conducted in Europe​; [23–29] two in America; [30, 31] one in Oceania​​​;​​​ [32] and one in Asia, [33] Table 2. The sample size varied from 55 to 3206, with children and adolescents from 6 to 18 years of age. Only in two studies did the female sex predominate; [30,33] however, most children and adolescents with obesity or overweight were male. (Table 2)

**Table 2:** Characteristics of the population and parameters evaluated

Reference	Country	Sample size Groups n=(%)	Sex M: F n=(%)	Age years (range) and *mean±SD according to sex	Parameters evaluated
**Lazzer S.**et al., **2009**	Italy	55 OB= 25 Non-OB=30	**M**:30(54.54) **F**:25(45.45) OB: **M**:12(21.8) **F**:13(23.6) Non-OB: **M**:18(32.7) **F**:12(21.9)	(8-12) OB = **M**: 9.7+0.5* **F**: 10.3+0.4* Non-OB = **M**:10.8+0.2* **F**:10.4+0.4*	Strength, Power (Maximal explosive power)
**Riddiford-Harland, DL.**et al., **2006**	Australia	86 OB= 43 Non-OB=43	**M**:50(58.1) **F**: 36(41.9) OB= **M**: 25 (29.1) **F**: 18(20.9) Non-OB: **M**: 25 (29.1)**F**: 18(20.9) ND***	OB: 8.4+0.5* Non-OB: 8.4+0.5*	Upper limb strength and lower limb strength and power
**He, H.**et al., **2019**	China	2283 OW/OB=7.05% NW=82.29% UW=12.66%	**M**:1032 (45.2)** F**:1251 (54.8) OW/OB: **M**:(9.98) **F**:(4.64) NW: **M** (75.78) **F**:(84.01) UW: **M**(14.24) **F**:(11.35)	(7-18)	Strength, power, flexibility
**Ervin, RB.** ***et a*** **l, 2014**	United States	1224 OB: 250, 19.0% OW: 214 (18.2%) UW/NW:760 (62.7%)	**M**: 607 (49.6) **F**: 617 (50.4) OB:** M:**105 (15.1) **F**:145 (22.8) OW: **M**: 118 (20.2)** F**:96 (16.3) UW/NW: **M**: 394 (60.8) **F**: 366 (60.8)	(6-15)	Upper and lower limb strength
**Karppanen, AK.**et al., **2012**	Finland	119 **M: **66 (55.46) OW: 30 (25.21) NW: 36 (30.25) **F**: 53 (44.53) OW: 24 (20.16) NW=29 (24.36)	**M**: 66 (55.5) **F**: 53 (44.5)	8 OW= **M**: 8.0 (0.4) **F**: 7.9 (0.4) NW= **M**: 8.0 (0.5) **F**: 8.1 (0.4)	Strength, power, velocity, coordination, balance, flexibility, agility, cardiovascular capacity, physical activity level (PAQ-C)
**Nunez-Gaunaurd, N.** ***et a*** **l, 2013**	United States	86 OB: 19= 22.09% OW: 20= 23.26% HW: 48= 55.81%	**M**: 47 (54.6) **F:** 39 (45.3) OB= **M: **10(11.6)**F**: 9(10.5) OW= **M**: 13(15.1)** F**: 7(8.1) HW= **M**: 24(27.9) **F:** 23(26.7)	OB: 10-14 11.9 (1.10) OW: 11-14 12.4 (0.093) HW: 11-15 12.3 (0.95)	Strength, gross and fine motor efficiency, cardiovascular capacity, MAP, physical activity level
**Deforche, B.**et al., **2003**	Belgium	3206 OB= **M**: 122 **F**: 108 Non-OB= **M**: 1524 **F**: 1452	**M**: 1646 (51.34) **F**: 1560 (48.65)	(12-18)	Strength, power, physical activity level, balance, cardiovascular capacity, coordination flexibility, agility, velocity
**Tokmakidis, S.**et al., **2006**	Greece	709 OB:105 (14.8%) OW:183 (25.8%) NW:421 (59.4%)	**M**: 381 (53.7) **F**: 328 (46.3)	(8-11) OB= **M:** 8.9+1.4* **F**: 8.6+1.5* OW= **M:** 9.1+1.6* F: 9.3+1.5* NW= **M:** 8.9+1.7* F: 8.8+1.6*	Strength, power, balance, cardiovascular capacity, coordination, flexibility, agility, velocity, endurance, speed
**Sachetti, R.**et al., **2012**	Italy	497 OB: (9.7%) OW: (24.3%) NW: (64.4%) 489 n= OB: 48 OW: 121 NW: 310	**M**:256 (51.5) **F**:241 (48.5) OB= **M**: 23 (9.0) **F**: 25 (10.4) OW= **M**: 65 (25.4) **F**: 56 (23.2)	(8-9) **M:** OB:23 OW:65 NW:155 **F:** OB:25 OW:56 NW:155 **EV: 8	Power, flexibility, coordination, agility, cardiovascular capacity, proprioception
**Castro-Piñero, J.**et al., **2019**	Spain	2778 OB:194 (7.0) OW:667 (24.0) NW:1750 (63.0)	**M**:1513 (54.4) **F**:1265 (45.5)	(6-17.9)	Power, strength
**Ceschia, A.**et al., **2015**	Italy	2408 OB: 166 OW: 441 NW: 1586	**M**: 1265 (52.5) **F:** 1146 (47.5) OB= **M:** (9.0) **F:** (7.4) OW= **M:**(19.3) **F:** (19.4) NW=** M:** (62.1) **F**: (64.2)	(7-11)	Strength, power, agility, velocity, cardiovascular capacity, flexibility, static balance

*Mean and standard deviation; **M**= males, **F**= female; UW= underweight, NW= healthy weight, non-OB= non obese, OW= overweight, OB= obesity; ND= not described; MAP= mean arterial pressure; **EV= eliminated values by authors; ***= matched to the experimental group for gender and age

### Muscle strength

The handgrip strength was higher in children and adolescents with overweight or obesity than in normal weight, especially in males [24, 30, 33]. Contrary, when upper limb strength was assessed in patients with overweight or obesity through a load of their weight (Bent arm hang test), the results were significantly lower compared to normal-weight patients, predominantly male [23, 25]. Similarly, in five articles, upper limb strength was assessed through pull-ups [30, 32]and push-up tests, [23, 31, 32] finding lower performance in the group with obesity.

Furthermore, the maximal strength of the knee extensor muscles was also higher in males with obesity participants than in normal weight [30]. In this regard, Castro-Piñero J. et al., 2009; ​​Deforche B. et al., 2003; Karppanen A K. et al., 2012; and Tokmakidis S. et al., 2006, found a lower number of sit-ups in the obesity group compared to those of normal weight or non-obese group, as viewed in Table 3 [23–25, 28]. In addition, one study [32] evaluated lower limb strength through the rising from a chair test, finding that participants with obesity were slower to get up from the chair than their counterparts. Moreover, Nunez-Gaunaurd N. et al., 2013, used timed tests for going up and down stairs, the Timed-Sit-to-Stand test and the Timed Up and Down Stairs test group with obesity as an indicator for lower limb strength, finding lower results in children with obesity compared to the group of participants with normal weight (OB: 1.8 ± 0.52; NW: 2.02 ± 0.52) [31]. Lastly, Lazzer S. et al., 2009 reported that the peak force of the lower limbs measured through the Explosive Ergometer was higher in participants with obesity compared with their counterparts non-obese showing a significant difference between male and female participants (boys: OB 975.8 ± 20.6; non-OB 867.4 ± 21.3; girls: OB 927.8 ± 21.8; non-OB 689.5 ± 25.1) [29] Table 3.

**Table 3:** Muscle strength

Reference	Assessment test	Muscle group assessed	Muscle strength result	
**Lazzer S.**et al., **2009**	Absolute peak force in both legs Explosive-Ergometer (EXER,University of Udine, Udine, Italy) (N)	Lower limbs	Boys: OB: 975.8± 20.6* Non obese: 867.4± 21.3*	Girls: OB: 927.8± 21.8* Non obese: 689.5± 25.1*
**Riddiford-Harland L.**et al., **2006**	Rising from a chair (s) (only 13 participants from each group completed the test)	Lower limbs	OB: 3.8±1.8* Leaners counterparts: 1.5 ±0.2*	
	Pull strenght (kg)†	Upper limbs	OB: 9.28 Non-obese: 8.71 OB: 9.6 ± 3.0 Non-OB: 8.8 ± 2.3	
	Push strenght (kg)†	Upper limbs	OB: 9.51 Non-obese: 8.71 OB: 9.3 ± 2.3 Non-OB: 8.8 ± 2.2	
**He H.**et al., **2019**	Hand grip strength (kg)	Upper limbs	Boys: OW/OB: 24.06 NW: 22.85 UW: 18.11	Girls: OW/OB:18.88 NW: 16.79 UW: 14.39
**Ervin RB.**et al., **2014**	Planks (s)	Abdominal and trunk	Boys: OB: 43.9 OW: 69.6 NW: 83.0	Girls: OB: 37.8 OW: 59.6 NW: 76.3
	Modified pull-ups in 3 categories according to the number of repetitions achieved (%)	Upper limbs	Boys: Zero OB: 37.7 OW: 14.5 NW: 6.2 1-4 OB: 35.2 OW: 37.8 NW: 29.7 Five or more OB: 27.1 OW: 47.7 NW: 64.1	Girls: Zero OB: 74.1 OW: 30.4 NW: 16.4 1-4 OB: 21.7 OW: 38.7 NW: 35.7 Five or more OB: 4.2 OW: 30.9 NW: 47.9
	Maximum right knee extension test (lb)		Boys: OB: 70.2 (31.8 kg) OW: 69.5 (31.5 kg) NW: 54.2 (24.6 kg)	Girls: OB: 70.6 (32.0 kg) OW: 62.5 (28.4 kg) NW: 52.8 (23.9 kg)
	Combined hand grip strength (lb)		Boys: OB: 109.6 (49.7 kg) OW: 106.6 (48.3 kg) NW: 93.7 (42.5 kg)	Girls: OB: 98.4 (44.7 kg) OW: 94.6 (42.9 kg) NW: 81.4 (36.9 kg)
**Karppanen AK.**et al., **2012**	Bent-arm hang (s)		Boys: OW: 1.8±2.5* NW: 13.6±11.3*	Girls: OW: 1.2±1.2* NW: 10.3±8.4*
	Sit-ups (repetitions in 30 s)	Abdominal and trunk	Boys: OW: 6.8±5.9* NW : 11.4± 5.6*	Girls: OW: 5.6±4.8* NW: 10.6 ± 5.3*
**Nunez-Gaunaurd N.**et al., **2013**	Abdominal curls (repetitions in 30 s)	Abdominal and trunk	OB: 5.4 ± 4.2* OW: 9.3± 5.26* HW: 9.7 ± 2.96*	
	Timed Sit-to-Stand test (steps in 1 min)	Lower limbs	OB: 23.1 ± 4.6* OW: 26.5 ± 5.1* HW: 27.25 ± 6.9*	
	Push-ups (repetitions in 30 s)	Upper limbs	OB: 8.8 ± 6.83* OW: 11.2 ± 6.71* HW: 10.86 ± 6.56*	
	Timed Up and Down Stairs Test (steps/s)	Lower limbs	OB: 1.8 ± 0.52 OW: 2.09 ± 0.59 HW: 2.02 ± 0.52	
**Deforche B.**et al., **2003**	Hand grip strength (kg)	Upper limbs	Boys: 12-13 years OB:31.0 ±7.5* Non-OB: 27.2± 7.0* 14-15 years OB: 41.7± 9.0* Non-OB: 37.4 ± 9.2* 16-18 years OB: 51.1 ± 8.1* Non-OB: 47.3 ±8.7*	Girls: 12-13 years OB: 26.8 ± 5.0* Non-OB:24.3 ± 5.5* 14-15 years OB: 31.5 ± 5.7* Non-OB: 29.1 ±5.4* 16-18 years OB: 33.3 ± 6.1* Non-OB: 31.1 ± 5.8*
	Bent-arm hang (s)	Upper limbs	Boys: 12-13 years OB: 2.2 ± 2.8* Non-OB: 17.6 ± 12.4* 14-15 years OB: 5.3 ± 5.2* Non-OB: 24.5 ± 14.3* 16-18 years OB: 6.1 ± 6.7* Non-OB: 32.7 ± 15.5*	Girls: 12-13 years OB: 1.0 ± 1.0* Non-OB: 10.0 ± 9.4* 14-15 years OB 1.1 ± 1.5* Non-OB: 10.8 ± 10.2* 16-18 years OB: 1.1 ± 1.1* Non-OB: 10.6 ± 10.7*
	Sit-ups (repetitions)	Abdominal and trunk	Boys: 12-13 years OB: 19.5 ±3.2* Non-OB: 24.1 ± 4.1* 14-15 years OB: 20.6±3.5* Non-OB: 24.9± 3.7* 16-18 years OB: 21.8±3.8* Non-OB: 25.8± 3.6*	Girls: 12-13 years OB: 15.4 ± 5.2* Non-OB: 20.5 ±3.9* 14-15 years OB: 17.8 ± 4.0* Non-OB: 20.8 ± 4.3* 16-18 years OB: 17.5 ± 4.4* Non-OB: 21.3 ± 4.0*
**Tokmakidis S.**et al., **2006**	Sit-ups (repetitions in 30 s)	Abdominal and trunk	Boys: OB: 14.6 OW: 17.7 NW: 19.0	Girls: OB: 11.6 OW: 15.7 NW: 17.5
**Castro-Piñero J.**et al.**2009**	Curl-ups (repetitions in 60 s) †	Abdominal and trunk	Boys: OB: 29.48 OW: 33.48 NW: 33.32 UW: 34.24	Girls: OB: 26.24 OW: 30.47 NW: 31.33 UW: 31.27
***Continue*** Table 3***…***	Sit-ups (repetitions in 30 s) †	Abdominal and trunk	Boys: OB: 17.57 OW: 20.53 NW: 21.31 UW: 21.25	Girls: OB: 16.51 OW: 18.43 NW: 19.28 UW: 19.18
	Bent arm hang (seconds) †	Upper limbs	Boys:OB: 3.06 OW: 5.90 NW: 12.9 UW: 17.04	Girls: OB: 1.00 OW: 1.74 NW: 5.24 UW: 5.35
	Push-ups (repetitions) †	Upper limbs	Boys: OB: 5.75 OW: 9.93 NW: 14.72 UW: 13.53	Girls: OB: 5.72 OW: 6.70 NW: 8.57 UW: 8.50
	Pull-ups (were not described according to BMI)			
**Ceschia A.**et al., **2015**	Hand grip strength (N)	Upper limbs	Clase of age I: OB: 118.0 ± 22.0* OW: 113.3 ± 25.5* NW: 110.1 ± 21.3* UW: 104.8 ± 22.5* Clase of age II: OB: 147.6 ± 31.4* OW: 142.6 ± 36.3* NW: 127.5 ± 24.9* UW: 118.7 ± 18.7*	Clase of age III: OB: 175.3 ± 37.2* OW: 154.4 ± 31.3* NW: 147.6 ± 34.8* UW: 139.0 ± 28.8* Clase of age IV: OB: 215.6 ± 38.8* OW: 190.6 ± 37.8* NW: 172.4 ± 38.5* UW: 146.6 ± 29.1* Clase of age V: OB: 229.2 ± 51.3* OW: 214.0 ± 51.6* NW: 198.6 ± 37.6* UW: 172.4 ± 35.0*

* Mean and standar deviation, s= seconds, kg=kilograms lb= pounds, N=Newtons, UW= Underweight, HW= Healthy weight, OW= Overweight, OB= Obesity, BOT2= Bruininks Oseretsky test second edition, †= values calculated by WebPlotDigitalizer- Copyright 2010-2021 Ankit Rohatgi

Some studies evaluated abdominal muscle strength, finding that the strength measured through plank and abdominal exercises was lower in the participants with obesity compared to those with normal weight and even those with overweight in both sexes, predominantly female [23, 30].

### Muscle power

Muscle power of the upper limbs was consistently higher in participants in the group with obesity [23, 26, 27, 32], especially in male participants. Only one study reported an increase in older age participants [26].

On the other hand, we found reduced muscle power in the lower limbs in the group with obesity compared to children and adolescents with normal weight, higher in male participants compared to females (Table 4) [23–28, 32, 33]. Only one study demonstrated greater muscle power in the lower limbs of the group of participants with obesity compared to overweight and normal weight with a predominance of males [29].

**Table 4:** Muscle power

Reference	Assessment test	Muscle group assessed	Muscle power result	
**Lazzer S.**et al., **2009**	Mechanical power peak (W)	Lower limbs	Boys: OB: 1281.5 ±95.9* Non-OB: 1076.2 ±81.2*	Girls: OB: 986.8±80.9* Non-OB: 754.8 ±78.1*
**Riddiford-Harland L.**et al., **2006**	Basketball throw(m)†	Upper limbs	OB: 3.39 Non-OB: 3.12	
	Vertical jump (cm)†	Lower limbs	OB: 22.22 Non-OB: 27.77	
	Standing long jump (cm)†	Lower limbs	OB: 90.32 Non-OB: 101.27	
**He H**, et al., **2019**	Vertical jump (evaluated by the squat jump test) (cm)	Lower limbs	Boys (mean): OW & OB :19.93 NW: 23.27 UW: 22.46	Girls (mean): OW & OB: 17.32 NW: 18.05 UW: 18.68
**Karppanen AK.**et al., **2012**	Standing broad jump (cm)	Lower limbs	Boys: OW: 110.2 ±15.0* NW 126.9 ±14.1*	Girls: OW: 102.7 ±15.7* NW: 115.7 ±15.5*
**Deforche B.**et al., **2003**	Standing-broad jump (cm)	Lower limbs	Boys: 12-13 years OB: 154.3 ± 17.8* Non-OB: 175.0 ± 19.2* 14-15 years OB: 173.1 ± 21.6* Non-OB: 193.8 ± 22.4* 16-18 years OB: 184.0 ± 20.8* Non-OB: 211.1 ± 22.3*	Girls: 12-13 years OB: 136.9 ± 14.6* Non-OB: 160.7 ± 17.8* 14-15 years OB: 142.3 ± 15.2* Non-OB: 165.8 ± 20.0* 16-18 years OB: 148.9 ± 17.4* Non-OB: 166.5 ± 20.5*
**Tokmakidis S.**et al., **2006**	Standing broad jump (cm)	Lower limbs	Boys: OB: 110.5 OW: 122 NW: 129	Girls: OB: 97.6 OW: 107.3 NW: 115.7
**Sacchetti R.**et al., **2012**	The 2 kg medicine-ball forward throw test (cm)	Upper limbs	Boys: OB: 391.7 ± 74.6* OW: 346.1 ±73.3* NW: 306.2 ± 61.0*	Girls: OB: 303.1 ± 42.0* OW: 285.3 ± 49.8* NW: 254.4 ± 43.2*
	Standing Long jump (cm)	Lower limbs	Boys: OB: 117.2 ±17.4* OW: 122.9 ±18.2* NW: 133.4 ±18.1*	Girls: OB: 102.2 ±16.5* OW 109.3 ±16.1* NW: 178.2 ±16.9*
**Castro-Piñero J.**et al., **2009**	Throw ball (m)†	Upper limbs	Boys: OB: 8.39 OW: 8.27 NW: 8.10 UW: 7.40	Girls: OB: 6.67 OW: 6.55 NW: 6.21 UW: 6.23
	Standing broad jump (m)†	Lower limbs	Boys: OB: 1.30 OW:1.42 NW: 1.52 UW: 1.51	Girls: OB: 1.19 OW: 1.24 NW: 1.31 UW: 1.43
	Vertical jump (cm)†	Lower limbs	Boys: OB: 24.14 OW: 26.28 NW: 29.19 UW: 27.71	Girls: OB: 20.52 OW: 22.47 NW: 24.04 UW: 23.61
**Ceschia A.**et al., **2015**	Throw back ball (m)	Upper limbs	Class of age I OB: 3.78 ± 0.81* OW: 3.66 ± 1.10* NW: 3.66 ± 1.15* UW: 3.58 ± 1.17* Class of age II OB: 4.63 ± 1.16* OW: 4.75 ± 1.09* NW: 4.63 ± 1.31* UW: 4.61 ± 1.18*	Class of age III OB: 5.38 ± 1.26* OW: 5.23 ± 1.21* NW: 5.02 ± 1.47* UW: 5.04 ± 1.52* Class of age IV OB: 6.61 ± 1.51* OW: 5.79 ± 1.19* NW: 5.67 ± 1.55* UW: 4.99 ± 1.53* Class of age V OB: 7.05 ± 1.07* OW: 6.88 ± 1.42* NW: 6.25 ± 1.26* UW: 5.59 ± 1.0*
	Long Jump (m)	Lower limbs	Class of age I OB: 0.75 ± 0.10 * OW: 0.86 ± 0.19* NW:0.92 ± 0.20* UW: 0.95 ± 0.17* Class of age II OB: 0.79 ± 0.22* OW: 0.91 ± 0.21* NW: 1.01 ± 0.18* UW: 1.05 ± 0.19*	Class of age III OB: 0.94±0.14* OW: 1.03±0.20* NW: 1.10±0.21* UW: 1.12±0.22* Class of age IV OB: 1.08±0.24* OW: 1.15±0.23* NW: 1.21±0.22* UW: 1.22±0.18* Class of age V OB: 1.13±0.10* OW: 1.20±0.20* NW: 1.26±0.25* UW: 1.25±0.18*

*Mean and standar deviation, m= meters, cm= centimeters, N=Newtons, W=mechanical power, UW= Underweight, HW= Healthy weight, Non-OB= Non-Obese, OW= Overweight, OB= Obesity, †=calculated by WebPlotDigitalizer- Copyright 2010-2021 Ankit Rohatgi

### Assessment tests or instruments

#### Muscle strength tests and Instruments

The most widely used measurement instrument for the evaluation of upper limb muscle strength was the hand dynamometer [24, 26, 30, 33] of different types (digital and mechanical), followed by indirect tests such as plank, pull-ups, or push-ups [30–32]. Bent arm hang as part of the EURO FIT battery [24, 25] or alone [23]. Abdominal muscle strength was assessed through abdominal curls [31] and sit-ups [23–25, 28]. For the evaluation of the strength of the lower limb muscles, the test used was the Explosive-Ergometer, (EXER, University of Udine, Udine, Italy), which assessed absolute peak strength of both limbs [29] and indirect tests such as rising from a chair test [32] the knee extension test [30] and Timed Sit-to-Stand test, and the Timed Up and Down Stair test (Table 3) [31].

#### Muscle power test and Instruments

The most used test to evaluate upper limb muscle power were the basketball throw, throwback ball, and forward throw tests [23, 26, 27, 32]. Conversely, lower limb muscle power was primarily assessed by jump tests, such as vertical jump, squat jump, standing broad jump, and long jump [23, 25–28, 32]. One study used a direct instrument, the Explosive Ergometer (EXER, University of Udine, Udine, Italy), to measure the absolute peak force of the lower extremities, an indicator of lower limb power. (Table 4) [29].

#### Quality of the evidence

The reporting methodological quality of the studies included showed a high risk of bias due to poor description of the measures taken to address the sample adequately, how the sample size was calculated, the strategies for performing the sampling, and whether the measurements were made through standardized and reliable instruments in all participants. (Table 1)

## Discussion

The present review allowed us to describe the absolute muscle strength and power in overweight or obese children and adolescents, as well as the most used tests and tools for its measurement, achieving our main objective. Among the most relevant results of our review, we found that the strength of the upper limbs measured through hand grip dynamometry with mechanical or digital tools was higher in children and adolescents with overweight and obesity compared to those of normal weight. Nevertheless, other tests, such as push-ups and bent arm hang in their original and modified versions, reported reduced upper limb strength in these groups. In addition, we found that muscle power measured through tests performed through jumps in its different modalities was consistently decreased in participants with obesity and overweight compared to those with normal weight regardless of age and sex, showing us poor performance of the muscles of the lower limbs in children with obesity or overweight.

Tomlinson 2016 and Musálek, 2020, reported similar findings to ours in people living with obesity, who found greater absolute strength compared to people with normal weight, although less strength per unit of body mass, probably explained by the intrinsic factors with affectation in neuromuscular activation and functional performance, previously described [14, 15, 34].

This review highlights poor muscular performance in overweight or obese participants when assessed through tests that involve repetitions (sit-ups, squats) or that support their body weight, such as planks, push-ups, pull-ups, and jumping jacks. However, we found a clear difference in strength assessed across tests such as grip strength and ball throwing, in which the tested participants did not carry their weight, performing better than normal-weight participants.

Another explanation to consider about the increase in strength in upper limbs in patients with overweight or obesity is the predominance of certain muscle fibres in upper limbs, for example, those of slow contraction (Type I) that are more resistant to fatigue, which explains in some way the elevated force found in the hand dynamometer test and ball throwing tests. On the contrary, in the muscles on the lower limb, which require greater strength, power, and speed for the execution of movements, the predominance of fast-twitch fibres (Type II) is noticeable, probably secondary to the structural changes due to obesity, the fatigue threshold and aerobic capacity reduced when body weight increases [13].

Although it has been reported that muscle strength depends on these intrinsic muscle factors mentioned, other biological, psychological, social, and environmental factors favor physical activity and, in turn, strength and muscle power [35].

In this sense, muscle strength is necessary to carry out daily life activities at each stage of life because muscle strength is relevant during childhood, a scenario in which they acquire new skills due to the nervous and musculoskeletal maturation process and the cardiorespiratory systems [36].

Environmental, psychological, and social factors are conditions that we should not ignore that surround individuals with overweight or obesity since it has been reported that when these factors are appropriate, the probability increases that the person performs a daily physical activity, with the improvement of their physical capacities, as well as their power and muscular strength [35] and that it reduces the risk of developing sarcopenia related to inactivity [37, 38].

An increase in physical activity of 60 to 200 min per week in prepubertal schoolchildren has been reported to be associated with increased muscular strength and endurance [39]. Muscular strength and endurance training positively affect all body systems by improving aerobic capacity and preventing disease development early on [40].

Despite the valuable information provided by our review, we acknowledge some limitations related to the great diversity and number of tests used to measure participants’ muscular strength and power. In addition to the above, some studies reported “adapting” the tests used in adults, which casts doubt on their validity in children and adolescents. Another limitation of this review was poor muscle performance in overweight or obese participants when assessed using tests involving repetitions (sit-ups, squats) or supporting their body weight, such as planks, push-ups, pull-ups, and jumping jacks” tests that could be complicated to perform in some individuals even with normal weight. However, despite the above, our review evidenced a consistent decrease in strength and power in overweight and obese children and adolescents in all studies included that could interfere with the treatment plan of these patients.

## Conclusions

There are significant differences in the power and absolute muscle strength of the upper or lower limbs between overweight and obese children and adolescents and those with normal weight, mainly when they are evaluated through tests through repetitions or when supporting their body weight.

### Electronic supplementary material

Below is the link to the electronic supplementary material.

Supplementary Material 1

Supplementary Material 2

## Data Availability

All data used and/or analyzed during this study are included in this published article.
