# Supplementary material for: Differences in the absolute muscle strength and power of children and adolescents with overweight or obesity: a systematic review
Source: BMC Pediatr. 2023 Sep 19;23:474. doi: 10.1186/s12887-023-04290-w (PMC10510195; doi:10.1186/s12887-023-04290-w)
Supplement: Supplementary file 1 — Supplementary Material 1 [file 12887_2023_4290_MOESM1_ESM.pdf]

Annex 1. Muscle strength tests

Test	Battery tests that include it	Explanation of the test	Illustration
Handgrip strength	PFAAT, ALPHA-FIT, EUROFIT, IPFT, ASSO-FTB , CPAFLA	Hand strength tests require a hand dynamometer. Various protocols exist for its application in physical fitness assessment depending on the battery test.	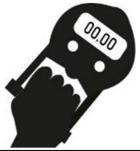
Modified Push-ups	NYPFP, NZFT, NFTP-PRC	The result is reported according to the number of repetitions performed without modifying the initial position and is determined by upper limb strength.	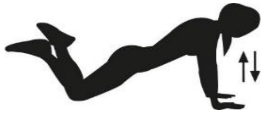
Bent arm hang	NYPFP, PFAAT, CPAFLA.	It measures the amount of time the individual can maintain the lifting position of the body on the bar, thereby giving a measure of upper trunk strength.	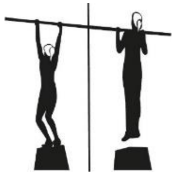
Curl up	NZFT, NYPFP, EUROFIT, NFTP-PCR, ASSO-FTB	The number of abdominal repetitions performed by the patient is measured.	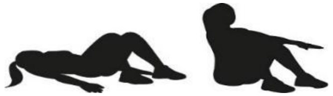
90° Push-ups	PCPF, NAPFA	The number of repetitions of push-ups performed by the patient is measured, assessing if he/she can complete at least five repetitions	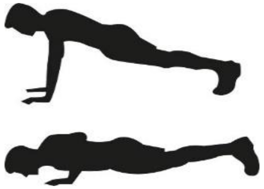
Squat 45°	PCPF	It measures the time the patient can hold the half-squat position, thereby estimating lower limb strength.	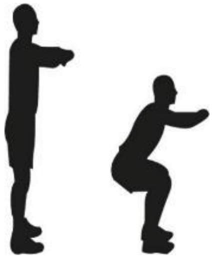

Continue Annex 1…			
Modified bent arm hang	EUROFIT, NFTP-PRC	The time that the patient can maintain the position is evaluated, thereby determining the strength of the upper limbs.	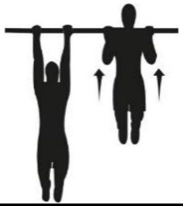
Modified pull-ups	FITNESSGRAM	Test the repeatability of pull-ups by lifting your chin above the bar. The push up test has replaced it.	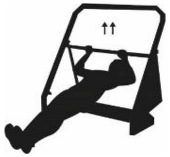
ALPHA-FIT: Assessing Levels of Physical Activity and Fitness; ASSO-FTB: Adolescents and Surveillance System for the Obesity prevention-Fitness Test Battery; CPAFLA: Canadian Physical Activity, Fitness, and Lifestyle Approach; EUROFIT: Eurofit battery tests; FITNESSGRAM: FITNESSGRAM youth fitness program; IPFT: International Physical Fitness Tests; NFTP-PRC: National Fitness Test Program in the Popular Republic China; NYPFP: National Youth Physical Program; NZFT: New Zealand Fitness Test; PCPF: President's Challenge: Physical Fitness; PFAAT: Physical Fitness and Athletic Ability Test.			

Annex 2. Muscle power tests

Test	Battery tests that include it	Explanation of the test	Illustration
Countermovement jump	NYPFP, NZFT	In this test, the individual is in an upright position with the hands on the waist, having to perform a vertical jump after a quick downward countermovement.	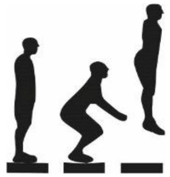
Vertical jump test	PFAAT	It measures the maximum height reached by the patient, jumping from an upright position and keeping the feet' soles flat on the ground.	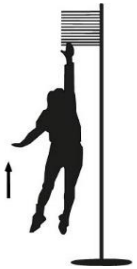
Standing long jump	PFAAT	It measures the muscular power of the lower limbs based on the distance achieved from the initial position.	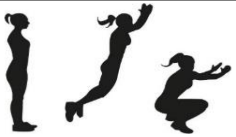
Standing broad jump	ALPHA-FIT, NFTP-PRC, NAPFA, CAPFLA, NYPFP, EUROFIT	It measures the muscular power of the lower limbs based on the distance achieved from the initial position.	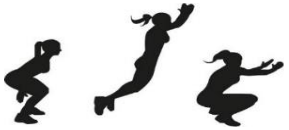
Ball throw	NZFT, PFAAT	Power of the trunk and upper limbs, measuring the distance of the thrown object, taking into account its weight.	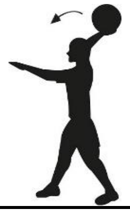
Shot put back throw	IPFT	Measures the distance reached by the patient during the throw in a sitting position	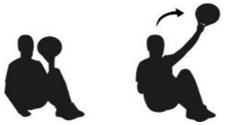

ALPHA-FIT: Assessing Levels of Physical Activity and Fitness; CPAFLA: Canadian Physical Activity, Fitness, and Lifestyle Approach; EUROFIT: Eurofit battery tests; IPFT: International Physical Fitness Tests; NAPFA: Singapore National Physical Fitness Award/Assessment; NFTP-PRC: National Fitness Test Program in the Popular Republic China; NYPFP: National Youth Physical Program; NZFT: New Zealand Fitness Test; PFAAT: Physical Fitness and Athletic Ability Test.
