# Supplementary material for: Differences in the absolute muscle strength and power of children and adolescents with overweight or obesity: a systematic review
Source: BMC Pediatr. 2023 Sep 19;23:474. doi: 10.1186/s12887-023-04290-w (PMC10510195; doi:10.1186/s12887-023-04290-w)
Supplement: Supplementary file 2 — Supplementary Material 2 [file 12887_2023_4290_MOESM2_ESM.docx]

**Annex. 1 Detailed information on the search strategy**

Table S1. Search terms contemplated in the Systematic Review.

| TERMS | TYPE | RESULTS |
| --- | --- | --- |
| Fitness | Text word | 114,348 |
| Muscle power | Text word | 26,484 |
| Obesity | Mesh | 428,373 |
| Muscle strength | Mesh | 82,792 |
| Physical fitness | Mesh | 55,403 |
| Assessment tools | Text word | 93,079 |
| Physical performance | Text word | 119,127 |
| Physical mobility | Text word | 87,151 |
| Physical fitness | Mesh | 55,403 |
| Physical endurance | Mesh | 47,392 |

Table S2. PubMed search strategy development.

| Search #1 | | |
| --- | --- | --- |
| ("overweight"[MeSH Terms] OR "overweight"[All Fields] OR "overweighted"[All Fields] OR "overweightness"[All Fields] OR "overweights"[All Fields]) AND ("obeses"[All Fields] OR "obesity"[MeSH Terms] OR "obesity"[All Fields] OR "obese"[All Fields] OR "obesities"[All Fields] OR "obesity s"[All Fields]) AND ("child"[MeSH Terms] OR "child"[All Fields] OR "children"[All Fields] OR "child s"[All Fields] OR "children s"[All Fields] OR "childrens"[All Fields] OR "childs"[All Fields]) AND ("muscular"[All Fields] AND ("perform"[All Fields] OR "performable"[All Fields] OR "performance"[All Fields] OR "performance s"[All Fields] OR "performances"[All Fields] OR "performative"[All Fields] OR "performatively"[All Fields] OR "performatives"[All Fields] OR "performativities"[All Fields] OR "performativity"[All Fields] OR "performed"[All Fields] OR "performer"[All Fields] OR "performer s"[All Fields] OR "performers"[All Fields] OR "performing"[All Fields] OR "performs"[All Fields])) AND ("muscle strength"[MeSH Terms] OR ("muscle"[All Fields] AND "strength"[All Fields]) OR "muscle strength"[All Fields]) AND ("physical fitness"[MeSH Terms] OR ("physical"[All Fields] AND "fitness"[All Fields]) OR "physical fitness"[All Fields]) | | |
| Results | Included filters | Results after the filters |
| 26 | Spanish  English | 25 |
| Table S3. Search strategies used in the selected databases. | | |
| **Epistemonikos** | | |
| children AND obesity AND overweight AND muscle strength OR muscle performance OR muscle fitness | | |
| Results | Included filters | Results after the filters |
| 37 | Primary study | 28 |
| **Tripdatabase** | | |
| children AND obesity AND overweight AND muscle strength OR muscle power | | |
| Results | Included filters | Results after the filters |
| 2398 | Primary study | 88 |
| **EBSCO** | | |
| overweight AND obese children AND muscular performance AND muscle strength, physical fitness | | |
| Results | Included filters | Results after the filters |
| 3280 | Journal sport sciences  Physical activity  Physical fitness  Muscle strength | 55 |
| **OVID** | | |
| obese children & muscle strenght & muscle power | | |
| 2533 | Niños de 0 a 18 años y de 6 a 12 años  Humanos  Inglés  Estudios observacionales  Profesionales de la salud  Artículos originales | 202 |
| **Science Direct** | | |
| obese children & muscle strength & muscle power | | |
| Results | Included filters | Results after the filters |
| 6053 | Medicine and Dentistry  Nursing and Health professions  Physical Medicine and Rehabilitation Clinics of North America  Journal of Science and Medicine in Sport | 75 |
| **NICE** | | |
| children AND obesity AND overweight AND muscle strength | | |
| Results | Included filters | Results after the filters |
| 8 | 0 | 8 |
| **BVS** | | |
| Results | Included filters | Results after the filters |
| children AND obesity AND overweight AND muscle strength OR muscle performance OR muscle fitness | | |
| 149 | Estudios observacionales, prevalencias y de tamizaje.  Español e inglés | 77 |
| **LILACS** | | |
| obese children, muscle strength, muscle power | | |
| Results | Included filters | Results after the filters |
| 14 | 0 | 14 |
